# Unilateral biportal endoscopic lumbar interbody fusion enhanced the recovery of patients with the lumbar degenerative disease compared with the conventional posterior procedures: A systematic review and meta-analysis

**DOI:** 10.3389/fneur.2022.1089981

**Published:** 2023-01-10

**Authors:** Honghao Yang, Fengqi Cheng, Yong Hai, Yuzeng Liu, Aixing Pan

**Affiliations:** Department of Orthopedic Surgery, Beijing Chao-Yang Hospital, Beijing, China

**Keywords:** unilateral biportal endoscopic lumbar interbody fusion, minimally-invasive transforaminal lumbar interbody fusion, posterior lumbar interbody fusion, lumbar degenerative disease, Enhanced Recovery After Surgery, neurosurgery

## Abstract

**Background:**

Minimally invasive endoscopic technique is an important component of Enhanced Recovery After Surgery (ERAS) protocol for neurosurgery. In recent years, unilateral biportal endoscopic lumbar interbody fusion (ULIF) has been used in the treatment of lumbar degenerative diseases (LDD). This study aims to investigate whether ULIF could enhance the recovery of patients with LDD compared with the conventional minimally invasive transforaminal lumbar interbody fusion (MI-TLIF) or posterior lumbar interbody fusion (PLIF).

**Methods:**

A comprehensive literature search was performed for relevant studies in PubMed, EMBASE, Web of Science, Cochrane Library database, China National Knowledge Internet, and Wanfang database. Surgical data, clinical outcomes, radiographic outcomes, and surgical complications were compared between patients with LDD who underwent ULIF and those who underwent conventional MI-TLIF or PLIF.

**Results:**

Notably, 12 studies, comprising 981 patients with LDD, were included. Of these patients, 449 underwent ULIF and 532 patients (355 MI-TLIF and 177 PLIF) were treated with conventional procedures. There was no significant difference in the fusion rate, cage subsidence rate, and surgical complications between the ULIF group and the MI-TLIF or PLIF group. Compared with MI-TLIF, the ULIF group presented a significantly reduced estimated blood loss (EBL) (WMD, −106.00; 95% CI −140.99 to −71.10, *P* < 0.001) and shorter length of hospital stay (LOS) (WMD, −1.27; 95% CI −1.88 to −0.66, *P* < 0.001); better short-term improvement in ODI (WMD, −2.12; 95% CI −3.53 to −0.72, *P* = 0.003) and VAS score for back pain (VAS-BP) (WMD, −0.86; 95% CI −1.15 to −0.58, *P* < 0.001) at 1 month post-operatively. Compared with PLIF, the ULIF group presented a significantly reduced EBL (WMD, −149.22; 95% CI −284.98 to −13.47, *P* = 0.031) and shorter LOS (WMD, −4.40; 95% CI −8.04 to −0.75, *P* = 0.018); better short-term improvement in VAS-BP (WMD, −1.07; 95% CI −1.77 to −0.38, *P* = 0.002) and VAS score for leg pain (VAS-LP) (WMD, −0.40; 95% CI −0.72 to −0.08, *P* = 0.014) at 1–2 week post-operatively; enhanced short- and long-term improvement in ODI at 1 month post-operatively (WMD, −3.12; 95% CI −5.72 to −0.53, *P* = 0.018) and the final follow-up (WMD, −1.97; 95% CI −3.32 to −0.62, *P* = 0.004), respectively.

**Conclusion:**

Compared with conventional MI-TLIF and PLIF, ULIF was associated with reduced EBL, shorter LOS, and comparable fusion rate as well as complication management. Compared with MI-TLIF, a better short-term improvement in VAS-BP and ODI was achieved by ULIF; compared with open PLIF, additional enhanced short-term improvement in VAS-LP and long-term improvement in ODI were observed in ULIF. ULIF could enhance the recovery of patients with LDD compared with conventional posterior procedures.

**Systematic trial registration:**

https://www.crd.york.ac.uk/prospero/display_record.php?RecordID=230695, CRD42021230695.

## Introduction

Lumbar degenerative disease (LDD), including lumbar spinal stenosis (LSS), lumbar disc herniation (LDH), and degenerative or isthmic lumbar spondylolisthesis (LS), has been one of the most prevalent and disabling spinal disorders that cause low back and leg pain, disability, and poor quality of life (1, 2). As a result, evolutions in both non-surgical and surgical treatment of LDD continue through the present day.

Among surgical procedures, lumbar interbody fusion is the gold standard for stabilizing spinal instability and decompressing neural elements (3). The most commonly used surgical approach is the posterior approach. Conventional posterior procedures include minimally invasive transforaminal lumbar interbody fusion (MI-TLIF) through microscopic tubular technique and open posterior lumbar interbody fusion (PLIF) (4). However, lumbar interbody fusion has been rated as one of the most painful procedures (5, 6). The main disadvantage of the conventional MI-TLIF or PLIF is the extensive paraspinal iatrogenic damage caused by dissection and retraction, which would induce the risk of chronic pain and delay patients' post-operative recovery and mobilization, placing a substantial economic burden on the public healthcare systems (7, 8). Therefore, there is a significant clinical and economic rationale for improving the management and outcomes of these conditions (9).

The concept of “fast-track” surgery was initiated by Kehlet in the 1990s and further developed as Enhanced Recovery After Surgery (ERAS) (10, 11). ERAS is a multidisciplinary and multimodal perioperative management approach that aims to improve surgical outcomes, reduce complications, and shorten the length of the hospital stay (12, 13). With the increasing application of ERAS protocols in neurosurgery, minimally invasive uniportal endoscopic technique has gained popularity as a key component for the management of lumbar interbody fusion (14–17). However, this technique was restricted by its vision and specific instruments. In recent years, the biportal endoscopic system and unilateral biportal endoscopic lumbar interbody fusion (ULIF) were developed to combine the advantages of conventional and endoscopic surgery (18–20). Through independent viewing and working channels, unrestricted vision, and ample operation space could be obtained while the posterior structure could be preserved. ULIF has been used to treat LDD; however, whether ULIF could enhance recovery compared with conventional procedures remains controversial.

The purpose of this systematic review and meta-analysis was to compare the surgical data, clinical outcomes, laboratory outcomes, radiographic outcomes, and surgical complications between ULIF and conventional MI-TLIF or PLIF for the treatment of LDD.

## Materials and methods

This study was designed according to the Preferred Reporting Items for Systematic Reviews and Meta-Analyses (PRISMA) guidelines and registered with PROSPERO (ID: CRD42021230695) (21, 22).

### Search strategy

PubMed, EMBASE, Web of Science, Cochrane Library database, China National Knowledge Internet, and Wanfang database were searched using the following terms: (fusion) AND [(((((UBE) OR (biportal endoscopic)) OR (unilateral biportal endoscopic)) OR (biportal endoscopic spinal surgery)) OR (unilateral laminotomy bilateral decompression)) OR (biportal endoscopy)].

The literature search was updated on 30 October 2022. Two reviewers (H.Y. and F.C.) independently screened the titles and abstracts, and any differences were settled by a discussion with a third reviewer (Y.L.).

### Surgical technique of ULIF

Under general anesthesia, the patients were placed in a prone position. C-arm fluoroscopy was performed to confirm the surgical level. The surface projection of the target bilateral pedicles and intervertebral space was marked on the skin. Two longitudinal skin incisions were made. Both the portals were 1.0 cm long, 3.0 cm apart from each other, and located 0.5 cm lateral to the ipsilateral spinous process. After the channel expanded through serial dilators, independent viewing and working channels were placed, and the submuscular operation space was formed on the surface of the lamina. A continuous fluid irrigation system with constant outflow was used. The paraspinal muscle attached to the lamina and articular process was detached by a stripper. Bipolar radiofrequency ablation could be applied for bleeding control. Laminectomy from the inferior edge of the cranial lamina to the superior edge of the caudal lamina and facetectomy for the medial edge of the articular process was performed using a power burr and gun forceps. Then, a flavectomy was performed to decompress the lumbar spinal canal and nerve root canals. The discectomy was operated under direct vision. After the endplate preparation, bone grafts and an interbody fusion cage were inserted. At last, bilateral percutaneous pedicle screw fixation was performed prior to the incision closure.

### Inclusion and exclusion criteria

The inclusion criteria are as follows: (1) patients diagnosed with lumbar degenerative diseases, including LSS, LDD, and LS of Meyerding grades I-II; (2) studies in which the intervention was ULIF; (3) studies comparing patients who underwent conventional MI-TLIF or PLIF; and (4) studies with the following outcomes: surgical data, clinical outcomes, laboratory outcomes, radiographic outcomes, and surgical complications.

The exclusion criteria are as follows: (1) studies that included patients with spinal tumors or infection; (2) studies that reported the outcomes of ULIF without comparison groups; (3) reviews, case reports, biomechanical analysis, and cadaveric research; (4) studies with no available full text; (5) duplicate publications; and (6) articles not published in English or Chinese.

### Assessment of study quality

Study quality was assessed independently by two reviewers (YH and AP) using the Newcastle-Ottawa scale (NOS) recommended by Cochrane Handbook version 5.2.0 (23). The level of evidence rating was assigned according to the published guidelines (24).

### Outcomes

Surgical data included estimated blood loss (EBL), operating time (ORT), length of hospital stay (LOS), and post-operative drainage. Clinical outcomes were Oswestry Disability Index (ODI) as well as visual analog scale (VAS) score for back pain (VAS-BP) and leg pain (VAS-LP) assessed at baseline, post-operatively, and the final follow-up. The excellent/good rate of surgical therapy according to the modified Macnab criteria was also evaluated at the final follow-up. Laboratory outcomes indicated serum creatine phosphokinase (CPK) and C-reactive protein (CRP) measured at baseline and 2 or 3 days post-operatively. Radiographic outcomes included cage subsidence rate and fusion rate at the final follow-up. Fusion was defined as the presence of bridging interbody trabecular bone using computed tomography scans or radiographs (25). Unplanned return to the operating room (OR) and surgical complications, including epidural hematoma, dural tear, surgical site infection, and neurologic deficits, were assessed during the perioperative period.

### Data extraction

Data extraction was performed independently by two reviewers (HY and FC). Demographic information, including age, sex, body mass index (BMI), diagnosis, operative level, and follow-up duration, was recorded. The data for 14 variables were extracted for analysis. Continuous outcomes included EBL, ORT, LOS, post-operative drainage, ODI, VAS-BP, VAS-LP, CPK, and CRP. Dichotomous outcomes included excellent/good rate of surgical therapy, cage subsidence rate, fusion rate, unplanned return to OR, and surgical complications.

### Data analysis

All statistical analyses were performed using the Stata version 15.1. Outcomes reported in at least two studies would be analyzed. For continuous outcomes, the weighted mean difference (WMD) or standard mean difference (SMD) was used to estimate the effect. The effect measure of dichotomous outcomes is displayed as a risk ratio (RR). The mean and standard deviation values of continuous outcomes or the counts and percentages of dichotomous outcomes for comparisons of data points are also displayed. The statistical heterogeneity among studies was evaluated using the I-square test and Cochran's *Q*-test. If the *I*^2^-value was <50% and the *P-*value was >0.10, a fixed-effects model was used. If the *I*^2^-value was >50% or the *P-*value was <0.10, a sensitivity analysis was applied to assess the impact of each study. If a source of potential heterogeneity could not be found, a random-effects model was used.

### Assessment of publication bias

Potential publication bias was assessed by applying Egger's test at a *P* < 0.10 level of significance (26). If publication bias was indicated, we further evaluated the number of missing studies by applying the “trim and fill” method and recalculated the pooled WMD, SME, or RR with the addition of those missing studies (27).

## Results

### Study selection

**Figure 1:**
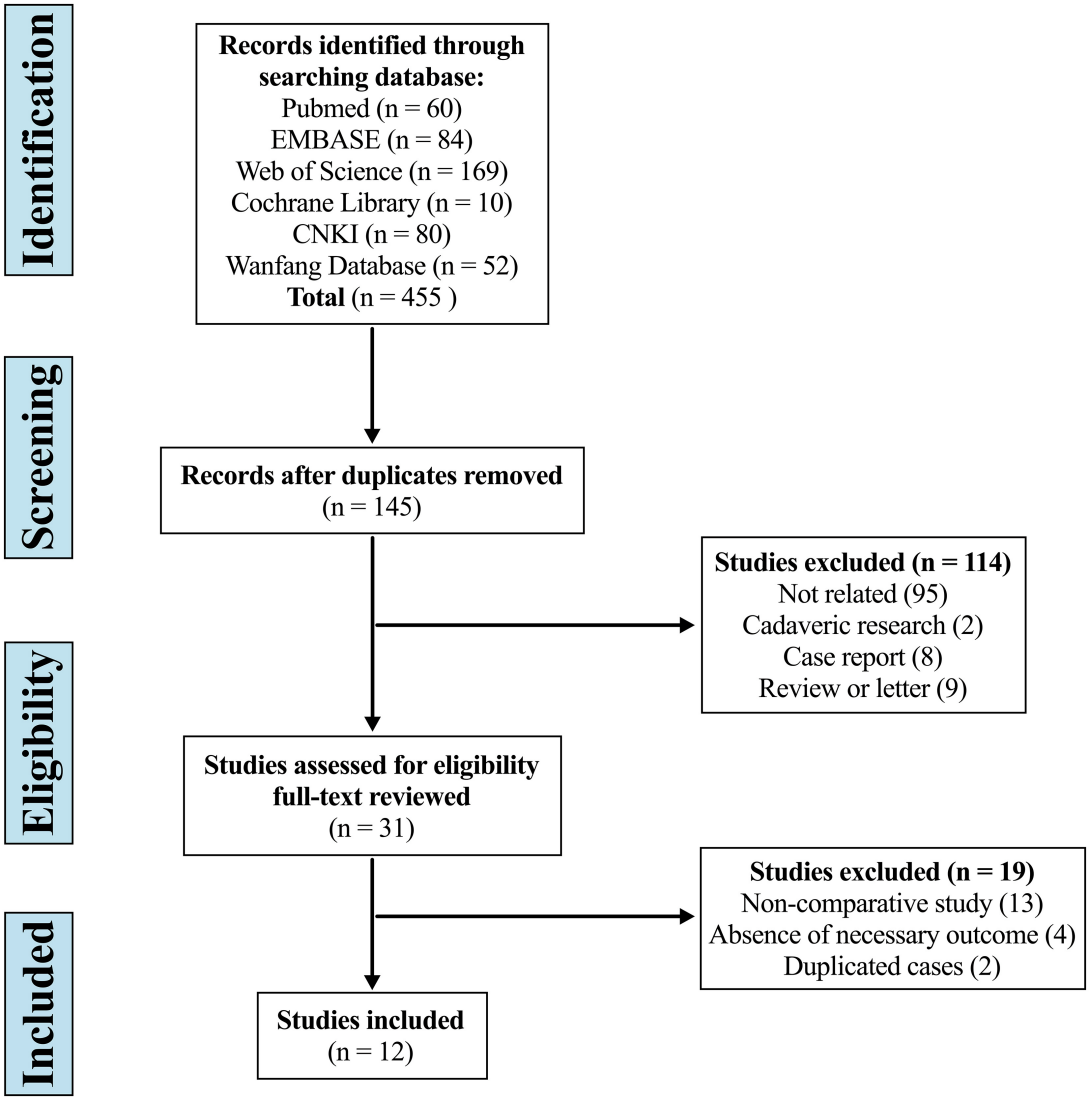
Flow diagram depicting the literature review, search strategy, and selection process.

The systematic search yielded 455 articles, of which 310 were duplicates, 114 were excluded by screening the title and abstract, and 19 were considered improper after full-text review. Eventually, 12 studies were included in this systematic review and meta-analysis (Figure 1) (28–39).

### Assessment of study quality and publication bias

The quality of the included studies was assessed using the Newcastle-Ottawa Scale (Table 1). Of the 12 studies included, eight were of high quality with scores of 8–9, and four were of moderate quality with scores of 7. The level of evidence was III for nine studies and IV for three studies. Publication bias was not detected for any variable.

**Table 1:** Quality assessment of studies according to Newcastle-Ottawa Scale (NOS).

**References**	**Year**	**Selection**	**Comparability**	**Exposure**	**Total score**
Heo and Park (33)	2019	3	2	3	8
Park et al. (38)	2019	4	2	3	9
Zhu et al. (31)	2021	4	2	2	8
Zhang et al. (37)	2021	3	2	2	7
Zhang et al. (36)	2021	3	2	2	7
Kim et al. (35)	2021	3	2	3	8
Kang et al. (34)	2021	4	2	3	9
Gatam et al. (32)	2021	4	2	2	8
Ma et al. (30)	2022	3	2	2	7
Liu et al. (39)	2022	4	2	3	9
Kong et al. (29)	2022	4	2	2	8
Jiang et al. (28)	2022	3	2	2	7

### Characteristics of included studies

Twelve studies, comprising 981 patients with LDD, were included. Of these patients, 449 underwent ULIF and 532 patients (355 MI-TLIF and 177 PLIF) were treated with conventional procedures. Characteristics of the included studies and patients are presented in Table 2. There were no significant differences at baseline between the ULIF group and MI-TLIF group in the patient's age (60.26 ± 9.26 years vs. 59.49 ± 9.90 years, *P* = 0.114), male-to-female ratio (0.75 vs. 0.86, *P* = 0.379), BMI (24.94 ± 3.24 kg/m^2^ vs. 25.32 ± 3.01 kg/m^2^, *P* = 0.111), diagnosis (*P* = 0.745), operative level (*P* = 0.382), ODI (58.75 ± 9.02 vs. 59.63 ± 8.21, *P* = 0.464), VAS-BP (6.43 ± 1.31 vs. 6.48 ± 1.25, *P* = 0.436), and VAS-LP (6.83 ± 1.85 vs. 6.79 ± 1.87, *P* = 0.246), CPK (*P* = 0.892), and CRP (*P* = 0.934). The duration of follow-up was 12.65 ± 2.74 months in the ULIF group and 13.25 ± 3.41 months in the MI-TLIF group (*P* = 0.098). Moreover, there were no significant differences at baseline between the ULIF group and PLIF group in the patient's age (60.50 ± 8.12 years vs. 61.58 ± 9.06 years, *P* = 0.574), male-to-female ratio (0.66 vs. 0.89, *P* = 0.500), BMI (24.56 ± 4.03 kg/m^2^ vs. 23.94 ± 3.83 kg/m^2^, *P* = 0.163), diagnosis (*P* = 0.521), operative level (*P* = 0.460), ODI (49.16 ± 9.10 vs. 45.95 ± 9.67, *P* = 0.129), VAS-BP (6.39 ± 1.29 vs. 6.50 ± 1.45, *P* = 0.076), and VAS-LP (5.98 ± 1.45 vs. 6.08 ± 1.41, *P* = 0.104). The duration of follow-up was 11.74 ± 3.49 months in the ULIF group and 12.63 ± 3.25 months in the PLIF group (*P* = 0.210).

**Table 2:** Characteristics of the included studies.

**References**	**Year**	**Design**	**Level of evidence**	**Group**	**Sample size**	**Age**	**Sex (M/F)**	**Diagnosis**	**Operative level**	**BMI (kg/m^2^)**	**FU (month)**
Heo and Park (33)	2019	Retrospective	III	ULIF	23	61.4 ± 9.4	7/16	LSS LS	L3/4 (3) L4/5 (17) L5/S1 (3)	NA	13.4 ± 2.5
				MI-TLIF	46	63.5 ± 10.5	19/27		L3/4 (4) L4/5 (29) L5/S1 (13)	NA	
Zhu et al. (31)	2021	Retrospective	III	ULIF	35	50.94 ± 12.12	16/19	LSS (19) LDH (7) LS (9)	L3/4 (0) L4/5 (28) L5/S1 (7)	NA	15.29 ± 1.98
				MI-TLIF	41	53.44 ± 14.37	19/22	LSS (21) LDH (13) LS (7)	L3/4 (2) L4/5 (25) L5/S1 (14)	NA	16.12 ± 2.59
Kim et al. (35)	2021	Retrospective	III	ULIF	32	70.5 ± 8.26	17/15	LS (32)	L2/3 (1) L3/4 (3) L4/5 (20) L5/S1 (8)	NA	27.2 ± 5.4
				MI-TLIF	55	67.3 ± 10.7	25/30	LS (55)	L2/3 (0) L3/4 (2) L4/5 (46) L5/S1 (7)	NA	31.5 ± 7.3
Kang et al. (34)	2021	Retrospective	IV	ULIF	47	66.87 ± 10.41	17/30	LSS LS	L2/3 (4) L3/4 (7) L4/5 (34) L5/S1 (20)	25.32 ± 3.15	14.5 ± 2.3
				MI-TLIF	32	66.38 ± 9.45	17/15		L2/3 (1) L3/4 (9) L4/5 (22) L5/S1 (11)	26.23 ± 3.26	15.78 ± 3.16
Gatam et al. (32)	2021	Retrospective	III	ULIF	72	55.1 ± 5.12	26/46	LS (72)	L3/4 (8) L4/5 (56) L5/S1 (8)	23.6 ± 3.67	≥ 12
				MI-TLIF	73	52.3 ± 6.13	28/45	LS (73)	L3/4 (10) L4/5 (48) L5/S1 (15)	24.8 ± 3.42	
Ma et al. (30)	2022	Retrospective	III	ULIF	32	58.81 ± 12.49	19/13	LSS (32)	L3/4 (1) L4/5 (23) L5/S1 (8)	24.96 ± 4.34	8.2 ± 1.5
				MI-TLIF	43	57.42 ± 9.67	26/17	LSS (32)	L3/4 (2) L4/5 (29) L5/S1 (12)	24.23 ± 3.37	
Kong et al. (29)	2022	Retrospective	III	ULIF	35	55.10 ± 7.75	13/22	LSS (12) LDH (15) LS (8)	L2/3 (1) L3/4 (5) L4/5 (17) L5/S1 (10) L4/S1 (2)	25.80 ± 1.80	≥ 6
				MI-TLIF	40	56.00 ± 8.00	18/22	LSS (15) LDH (9) LS (16)	L1/2 (1) L2/3 (4) L3/4 (7) L4/5 (15) L5/S1 (12) L4/S1 (1)	26.00 ± 2.00	
Jiang et al. (28)	2022	Retrospective	IV	ULIF	25	63.28 ± 8.51	9/16	LSS (25)	L4/5 (24) L5-S1 (1)	NA	≥ 3
				MI-TLIF	25	59.68 ± 10.38	8/17	LSS (25)	L4/5 (23) L5-S1 (2)	NA	
Park et al. (38)	2019	Retrospective	III	ULIF	71	68.00 ± 8.00	26/45	LSS (7) LDH (2) LS (62)	L3/4 (13) L4/5 (50) L5/S1 (8)	NA	17.1 ± 4.9
				PLIF	70	66.00 ± 9.00	20/50	LSS (11) LDH (2) LS (57)	L3/4 (8) L4/5 (56) L5/S1 (6)	NA	20.4 ± 7.2
Zhang et al. (37)	2021	Retrospective	IV	ULIF	21	58.90 ± 9.20	14/7	LSS LDS	NA	22.70 ± 5.90	≥ 6
				PLIF	35	62.80 ± 10.40	18/17			23.90 ± 6.20	
Zhang et al. (36)	2021	Retrospective	III	ULIF	29	51.14 ± 6.85	17/12	LDH (29)	NA	24.69 ± 3.16	≥ 12
				PLIF	39	53.92 ± 7.16	26/13	LDH (39)		23.84 ± 2.97	
Liu et al. (39)	2022	Prospective	III	ULIF	27	63.89 ± 8.44	12/15	LSS (7) LDH (14) LS (6)	L3/4 (4) L4/5 (18) L5/S1 (5)	24.91 ± 3.03	11.67 ± 5.05
				PLIF	33	63.70 ± 9.69	13/20	LSS (9) LDH (17) LS (7)	L3/4 (7) L4/5 (20) L5/S1 (6)	24.02 ± 2.32	12.15 ± 4.18

ULIF indicates unilateral biportal endoscopic lumbar interbody fusion; MI-TLIF, minimally invasive transforaminal lumbar interbody fusion; PLIF, posterior lumbar interbody fusion; LSS, lumbar spinal stenosis; LDH, lumbar disc herniation; LS, lumbar spondylolisthesis; BMI, body mass index; FU, follow-up; NA, not available.

### Surgical data

#### Estimated blood loss

##### ULIF vs. MI-TLIF

**Figure 2:**
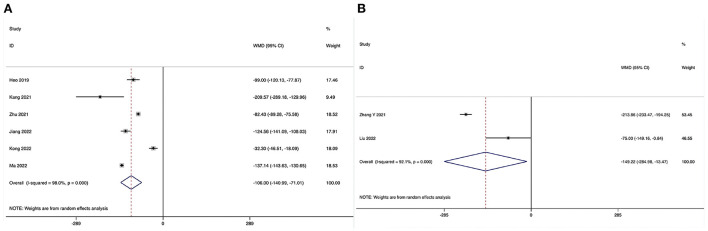
Forest plot of the estimated blood loss. **(A)** ULIF vs. MI-TLIF; **(B)** ULIF vs. PLIF.

Estimated blood loss could be obtained in six studies (28–31, 33, 34), and significant heterogeneity was detected (*I*^2^ = 98.0%, *P* < 0.001). The pooled results revealed significantly reduced EBL in the ULIF group compared with that in the MI-TLIF group (WMD, −106.00; 95% CI −140.99 to −71.10, *P* < 0.001; Table 3, Figure 2A).

**Table 3:** The pooled outcomes between ULIF and MI-TLIF group.

**Outcomes**	**Included studies**	ULIF^*^	MI-TLIF^*^	**WMD/SMD or RR**	**95% CI**		***P* Effect**	**Heterogeneity**	
								***I** ^2^*	***P***
**Surgical data**									
EBL	6	197	227	−106.00	−140.99	−71.10	<0.001	98.0%	<0.001
ORT	7	229	282	22.91	10.60	35.23	<0.001	92.8%	<0.001
LOS	6	206	236	−1.27	−1.88	−0.66	<0.001	66.4%	0.011
Post-operative drainage	3	104	100	−47.98	−68.15	−27.81	<0.001	89.2%	<0.001
**Clinical outcomes**									
**ODI**									
ODI at 1–2 week post-op	4	164	194	−4.70	−9.13	−0.27	0.038	92.2%	<0.001
ODI at 1 month post-op	3	107	97	−2.12	−3.53	−0.72	0.003	40.1%	0.188
ODI at 3 month post-op	5	196	237	−1.49	−2.77	−0.22	0.022	43.7%	0.130
ODI at 6 month post-op	3	151	148	−1.07	−4.00	1.86	0.473	70.2%	0.035
ODI at finial follow-up	8	301	355	−0.23	−0.69	0.24	0.346	11.2%	0.343
**VAS-BP**									
VAS-BP at 1–2 day post-op	2	95	119	−1.22	−1.30	−1.13	<0.001	33.0%	0.222
VAS-BP at 1–2 week post-op	2	67	96	−1.08	−1.50	−0.65	<0.001	0.0%	0.893
VAS-BP at 1 month post-op	4	149	168	−0.86	−1.15	−0.58	<0.001	36.5%	0.193
VAS-BP at 6 month post-op	2	119	105	−0.03	−0.37	0.30	0.853	0.0%	0.383
VAS-BP at finial follow-up	6	244	287	−0.12	−0.25	0.01	0.069	0.0%	0.995
**VAS-LP**									
VAS-LP at 1–2 week post-op	2	67	96	−0.20	−0.56	0.16	0.281	0.0%	1.000
VAS-LP at 1 month post-op	4	149	168	−0.15	−0.34	0.03	0.100	0.0%	0.592
VAS-LP at 6 month post-op	2	119	105	0.49	−0.02	1.00	0.059	0.0%	0.710
VAS-LP at finial follow-up	6	244	287	−0.02	−0.17	0.13	0.843	0.0%	0.563
Excellent/good rate	3	86.9% (86/99)	86.3% (120/139)	1.00	0.91	1.11	0.951	0.0%	0.856
**Laboratory outcomes**									
CPK	2	72	57	−1.15	−1.86	−0.45	0.001	69.0%	0.057
CRP	2	72	57	−1.21	−1.59	−0.83	<0.001	42.3%	0.188
**Radiographic outcomes**									
Fusion rate	7	89.8% (264/294)	87.7% (299/341)	1.02	0.96	1.07	0.545	0.0%	0.973
Cage subsidence	3	0.8% (1/127)	4.3% (7/162)	0.34	0.08	1.46	0.146	0.0%	0.498
Unplanned return to OR	4	1.1% (2/174)	1.9% (4/206)	0.76	0.20	2.93	0.687	0.0%	0.689
**Surgical complications**									
Overall	7	6.9% (19/276)	6.7% (22/330)	0.96	0.53	1.74	0.896	0.0%	0.992
Epidural hematoma	5	2.9% (5/172)	2.3% (5/214)	1.19	0.37	3.83	0.775	0.0%	0.951
Dural tear	5	4.8% (10/209)	2.1% (5/234)	2.08	0.76	5.74	0.156	0.0%	0.850
Surgical site infection	5	0.0% (0/209)	2.6% (6/234)	0.34	0.09	1.33	0.120	0.0%	0.985
Neurologic deficits	3	3.5% (4/114)	4.7% (6/128)	0.74	0.22	2.50	0.632	0.0%	0.721

* n or incidence (events/total).

ULIF, unilateral biportal endoscopic lumbar interbody fusion; MI-TLIF, minimally invasive transforaminal lumbar interbody fusion; WMD, weighted mean difference; SMD, standard mean difference; RR, risk ratio; CI, confidence interval; EBL, estimated blood loss; ORT, operating time; LOS, length of hospital stay; ODI, Oswestry Disability Index; VAS-BP, Visual Analog Scale score for back pain; VAS-LP, Visual Analog Scale score for leg pain; CPK, creatine phosphokinase; CRP, C-reactive protein; OR, operating room.

##### ULIF vs. PLIF

Estimated blood loss could be obtained in two studies (36, 39), and significant heterogeneity was detected (*I*^2^ = 92.1%, *P* < 0.001). The pooled results revealed significantly reduced EBL in the ULIF group compared with that in the PLIF group (WMD, −149.22; 95% CI −284.98 to −13.47, *P* = 0.031; Table 4, Figure 2B).

**Table 4:** The pooled outcomes between ULIF and PLIF group.

**Outcomes**	**Included studies**	ULIF^*^	PLIF^*^	**WMD/ SMD or RR**	**95% CI**		***P* effect**	**Heterogeneity**	
								***I** ^2^*	***P***
**Surgical data**									
EBL	2	56	72	−149.22	−284.98	−13.47	0.031	92.1%	<0.001
ORT	4	148	177	48.30	26.07	70.54	<0.001	94.7%	<0.001
LOS	3	77	107	−4.40	−8.04	−0.75	0.018	96.9%	<0.001
Post-operative drainage	3	77	107	−139.84	−216.22	−63.47	<0.001	95.5%	<0.001
**Clinical outcomes**									
**ODI**									
ODI at 1–2 week post-op	2	56	72	−3.40	−4.02	−2.78	<0.001	9.6%	0.293
ODI at 1 month post-op	2	48	68	−3.12	−5.72	−0.53	0.018	0.0%	0.551
ODI at finial follow-up	4	138	177	−1.97	−3.32	−0.62	0.004	37.7%	0.186
**VAS-BP**									
VAS-BP at 1–2 week post-op	2	88	103	−1.07	−1.77	−0.38	0.002	78.9%	0.030
VAS-BP at finial follow-up	3	117	142	−0.17	−0.37	0.04	0.114	0.0%	0.574
**VAS-LP**									
VAS-LP at 1–2 week post-op	2	88	103	−0.40	−0.72	−0.08	0.014	0.0%	0.465
VAS-LP at finial follow-up	3	117	142	0.01	−0.20	0.22	0.937	29.9%	0.240
Excellent/good rate	2	83.3% (40/48)	85.3% (58/68)	0.97	0.82	1.14	0.709	0.0%	0.561
**Radiographic outcomes**									
Fusion rate	2	94.3% (83/88)	90.3% (93/103)	1.04	0.96	1.13	0.296	0.0%	0.690
**Surgical complications**									
Overall	4	6.8% (10/148)	5.1% (9/177)	1.29	0.56	2.95	0.553	0.0%	0.429
Dural tear	4	4.7% (7/148)	2.8% (5/177)	1.68	0.57	4.92	0.344	0.0%	0.762
Neurologic deficits	2	1.1% (1/92)	1.0% (1/105)	1.25	0.20	7.64	0.811	28.5%	0.237

* n or incidence (events/total).

ULIF, unilateral biportal endoscopic lumbar interbody fusion; PLIF, posterior lumbar interbody fusion; WMD, weighted mean difference; SMD, standard mean difference; RR, risk ratio; CI, confidence interval; EBL, estimated blood loss; ORT, operating time; LOS, length of hospital stay; ODI, Oswestry Disability Index; VAS-BP, Visual Analog Scale score for back pain; VAS-LP, Visual Analog Scale score for leg pain.

#### Operating time

##### ULIF vs. MI-TLIF

**Figure 3:**
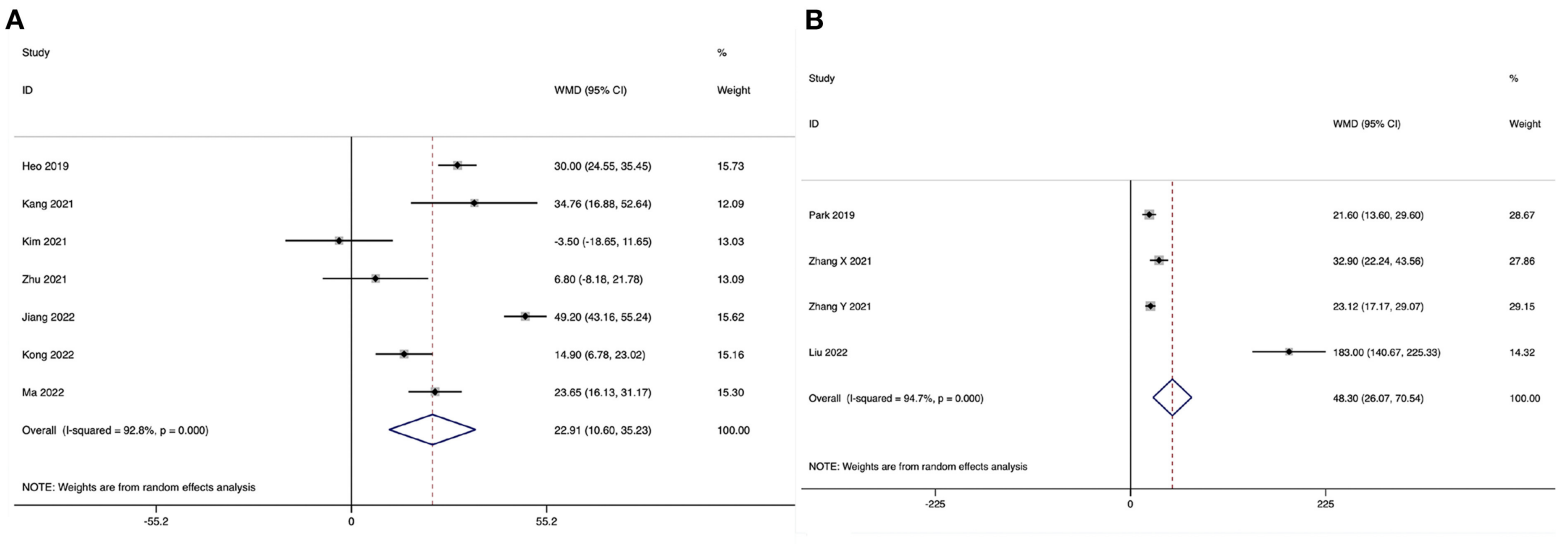
Forest plot of the operating time. **(A)** ULIF vs. MI-TLIF; **(B)** ULIF vs. PLIF.

Operating time could be obtained in seven studies (28–31, 33–35), and significant heterogeneity was detected (*I*^2^ = 92.8%, *P* < 0.001). The pooled results revealed significantly prolonged ORT in the ULIF group compared with that in the MI-TLIF group (WMD, 22.91; 95% CI 10.60–35.23, *P* < 0.001; Figure 3A).

##### ULIF vs. PLIF

Operating time could be obtained in four studies (36–39), and significant heterogeneity was detected (*I*^2^ = 94.7%, *P* < 0.001). The pooled results revealed significantly prolonged ORT in the ULIF group compared with that in the PLIF group (WMD, 48.30; 95% CI 26.07–70.54, *P* < 0.001; Figure 3B).

#### Length of hospital stay

##### ULIF vs. MI-TLIF

**Figure 4:**
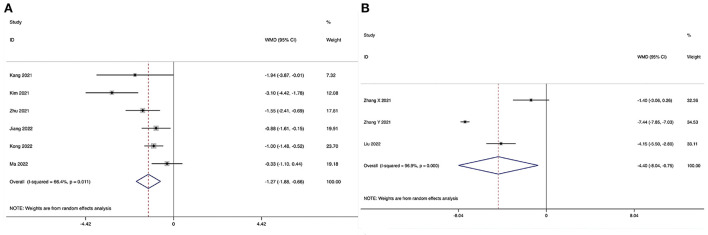
Forest plot of the length of hospital stay. **(A)** ULIF vs. MI-TLIF; **(B)** ULIF vs. PLIF.

The length of hospital stay could be obtained in six studies (28–31, 34, 35), and significant heterogeneity was detected (*I*^2^ = 66.4%, *P* = 0.011). The pooled results revealed significantly reduced LOS in the ULIF group compared with that in the MI-TLIF group (WMD, −1.27; 95% CI −1.88 to −0.66, *P* < 0.001; Figure 4A).

##### ULIF vs. PLIF

The length of hospital stay could be obtained in three studies (36, 37, 39), and significant heterogeneity was detected (*I*^2^ = 96.9%, *P* < 0.001). The pooled results revealed significantly reduced LOS in the ULIF group compared with that in the PLIF group (WMD, −4.40; 95% CI −8.04 to −0.75, *P* = 0.018; Figure 4B).

#### Post-operative drainage

##### ULIF vs. MI-TLIF

**Figure 5:**
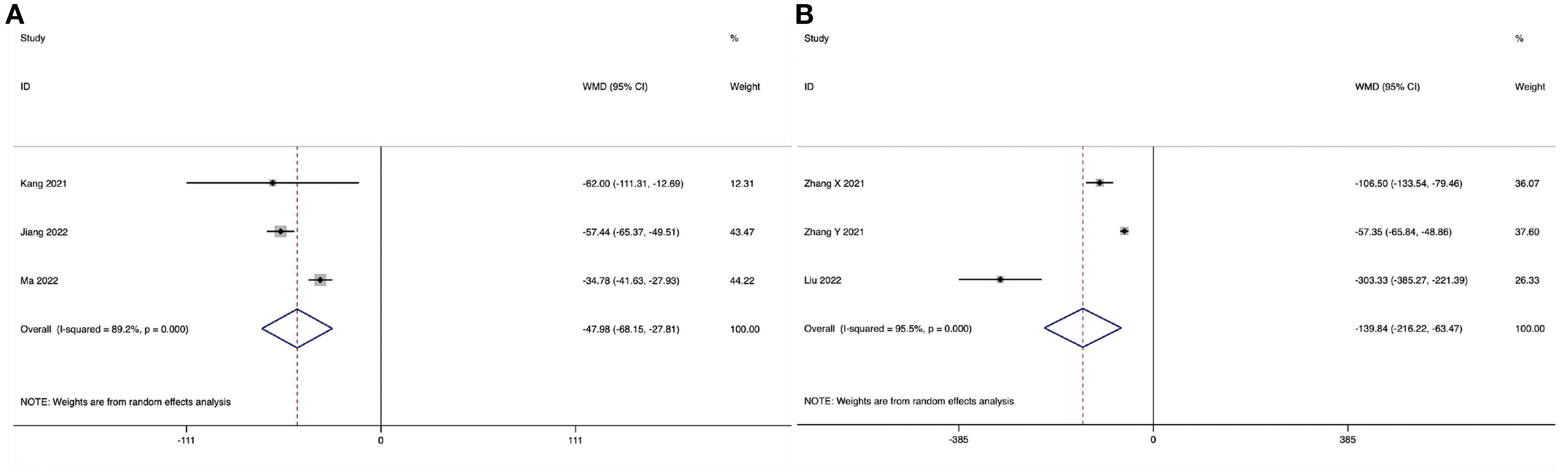
Forest plot of the post-operative drainage. **(A)** ULIF vs. MI-TLIF; **(B)** ULIF vs. PLIF.

Post-operative drainage could be obtained in three studies (28, 30, 34), and significant heterogeneity was detected (*I*^2^ = 89.2%, *P* < 0.001). The pooled results revealed significantly reduced post-operative drainage in the ULIF group compared with that in the MI-TLIF group (WMD, −47.98; 95% CI −68.15 to −27.81, *P* < 0.001; Figure 5A).

##### ULIF vs. PLIF

Post-operative drainage could be obtained in three studies (36, 37, 39), and significant heterogeneity was detected (*I*^2^ = 95.5%, *P* < 0.001). The pooled results revealed significantly reduced post-operative drainage in the ULIF group compared with that in the PLIF group (WMD, −139.84; 95% CI −216.22 to −63.47, *P* < 0.001; Figure 5B).

### Clinical outcomes

#### Oswestry disability index

##### One to two weeks post-operatively

###### ULIF vs. MI-TLIF.

The Oswestry Disability Index at 1–2 weeks post-operatively could be obtained in four studies (28, 31, 32, 35), and significant heterogeneity was detected (*I*^2^ = 92.2%, *P* < 0.001). The pooled results revealed significantly lower ODI at 1–2 weeks post-operatively in the ULIF group compared with that in the MI-TLIF group (WMD, −4.70; 95% CI −9.13 to −0.27, *P* = 0.038).

###### ULIF vs. PLIF.

The Oswestry Disability Index at 1–2 weeks post-operatively could be obtained in two studies (36, 39), and no significant heterogeneity was detected (*I*^2^ = 9.6%, *P* = 0.293). The pooled results revealed significantly lower ODI at 1–2 weeks post-operatively in the ULIF group compared with that in the PLIF group (WMD, −3.40; 95% CI −4.02 to −2.78, *P* < 0.001).

##### One month post-operatively

###### ULIF vs. MI-TLIF.

The Oswestry Disability Index at 1 month post-operatively could be obtained in three studies (28, 29, 34), and no significant heterogeneity was detected (*I*^2^ = 40.1%, *P* = 0.188). The pooled results revealed significantly lower ODI at 1 month post-operatively in the ULIF group compared with that in the MI-TLIF group (WMD, −2.12; 95% CI −3.53 to −0.72, *P* = 0.003).

###### ULIF vs. PLIF.

The Oswestry Disability Index at 1 month post-operatively could be obtained in two studies (37, 39), and no substantial heterogeneity was detected (*I*^2^ = 0.0%, *P* = 0.551). The pooled results revealed significantly lower ODI at 1 month post-operatively in the ULIF group compared with that in the PLIF group (WMD, −3.12; 95% CI −5.72 to −0.53, *P* = 0.018).

##### The third month post-operatively

###### ULIF vs. MI-TLIF.

The Oswestry Disability Index at 3 month post-operatively could be obtained in five studies (28, 30–32, 35), and no significant heterogeneity was detected (*I*^2^ = 43.7%, *P* = 0.130). The pooled results revealed significantly lower ODI at 3 month post-operatively in the ULIF group compared with that in the MI-TLIF group (WMD, −1.49; 95% CI −2.77 to −0.22, *P* = 0.022).

##### The sixth month post-operatively

###### ULIF vs. MI-TLIF.

The Oswestry Disability Index at 6 month post-operatively could be obtained in three studies (30, 32, 34), and significant heterogeneity was detected (*I*^2^ = 70.2%, *P* = 0.035). The pooled results revealed no significant difference in ODI at 6 month post-operatively between the ULIF group and the MI-TLIF group (WMD, −1.07; 95% CI −4.00–1.86, *P* = 0.473).

##### Final follow-up

###### ULIF vs. MI-TLIF.

**Figure 6:**
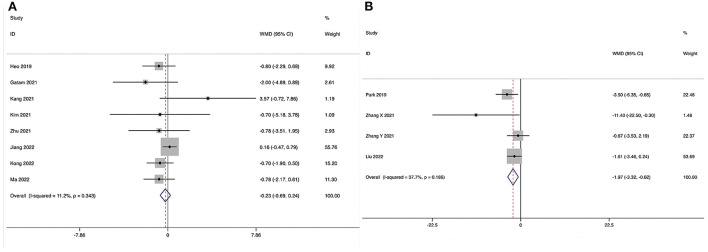
Forest plot of the Oswestry Disability Index at the final follow-up. **(A)** ULIF vs. MI-TLIF; **(B)** ULIF vs. PLIF.

The Oswestry Disability Index at the final follow-up could be obtained in eight studies (28–35), and no significant heterogeneity was detected (*I*^2^ = 11.2%, *P* = 0.343). The pooled results revealed no significant difference in ODI at the final follow-up between the ULIF group and the MI-TLIF group (WMD, −0.23; 95% CI −0.69–0.24, *P* = 0.346; Figure 6A).

###### ULIF vs. PLIF.

The Oswestry Disability Index at the final follow-up could be obtained in four studies (36–39), and no significant heterogeneity was detected (*I*^2^ = 37.7%, *P* = 0.186). The pooled results revealed significantly lower ODI at the final follow-up in the ULIF group compared with that in the PLIF group (WMD, −1.97; 95% CI −3.32 to −0.62, *P* = 0.004; Figure 6B).

### Visual Analog Scale score for back pain

#### One to two days post-operatively

##### ULIF vs. MI-TLIF

The Visual Analog Scale score for back pain at 1–2 days post-operatively could be obtained in two studies (32, 33), and no significant heterogeneity was detected (*I*^2^ = 33.0%, *P* = 0.222). The pooled results revealed significantly lower VAS-BP at 1–2 days post-operatively in the ULIF group compared with that in the MI-TLIF group (WMD, −1.22; 95% CI −1.30 to −1.13, *P* < 0.001).

#### One to two weeks post-operatively

##### ULIF vs. MI-TLIF

The Visual Analog Scale score for back pain at 1–2 weeks post-operatively could be obtained in two studies (31, 35), and no substantial heterogeneity was detected (*I*^2^ = 0.0%, *P* = 0.893). The pooled results revealed significantly lower VAS-BP at 1–2 weeks post-operatively in the ULIF group compared with that in the MI-TLIF group (WMD, −1.08; 95% CI −1.50 to −0.65, *P* < 0.001).

##### ULIF vs. PLIF

The Visual Analog Scale score for back pain at 1–2 weeks post-operatively could be obtained in two studies (38, 39), and significant heterogeneity was detected (*I*^2^ = 78.9%, *P* = 0.030). The pooled results revealed significantly lower VAS-BP at 1–2 weeks post-operatively in the ULIF group compared with that in the PLIF group (WMD, −1.07; 95% CI −1.77 to −0.38, *P* = 0.002).

#### One month post-operatively

##### ULIF vs. MI-TLIF

The Visual Analog Scale score for back pain at 1 month post-operatively could be obtained in four studies (29, 31, 34, 35), and no significant heterogeneity was detected (*I*^2^ = 36.5%, *P* = 0.193). The pooled results revealed significantly lower VAS-BP at 1 month post-operatively in the ULIF group compared with that in the MI-TLIF group (WMD, −0.86; 95% CI −1.15 to −0.58, *P* < 0.001).

#### The sixth month post-operatively

##### ULIF vs. MI-TLIF

The Visual Analog Scale score for back pain at 6 month post-operatively could be obtained in two studies (32, 34), and no substantial heterogeneity was detected (*I*^2^ = 0.0%, *P* = 0.383). The pooled results revealed no significant difference in VAS-BP at 6 month post-operatively between the ULIF group and the MI-TLIF group (WMD, −0.03; 95% CI −0.37–0.30, *P* = 0.853).

#### Final follow-up

##### ULIF vs. MI-TLIF

**Figure 7:**
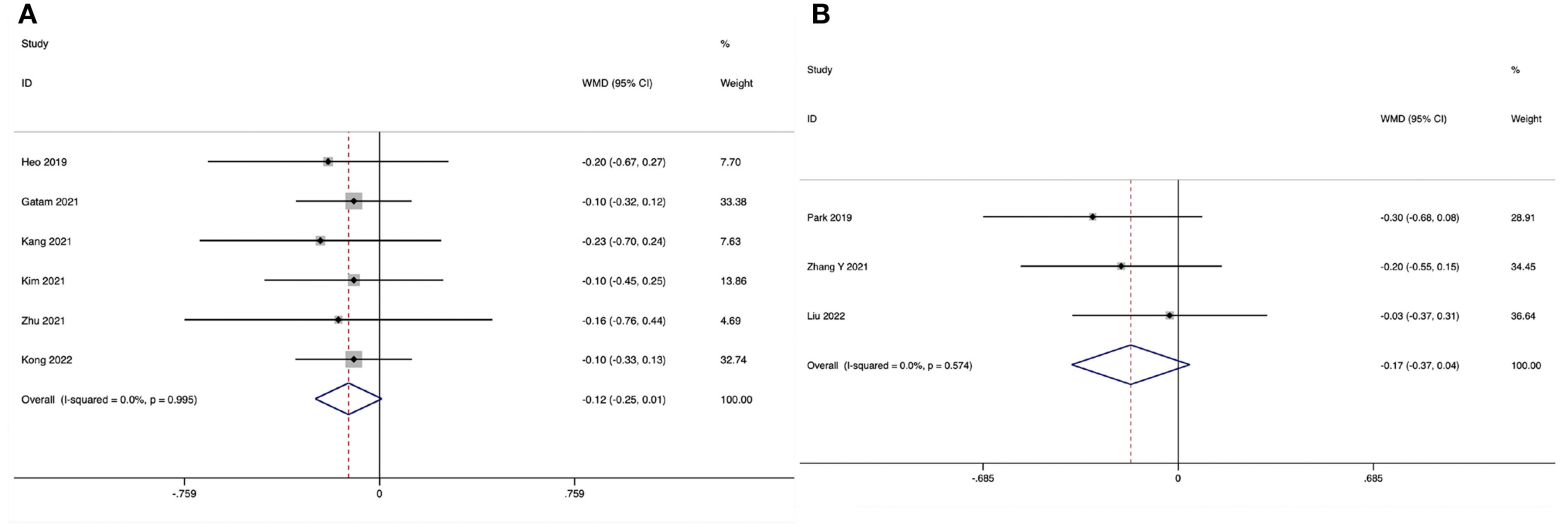
Forest plot of the Visual Analog Scale score for back pain at the final follow-up. **(A)** ULIF vs. MI-TLIF; **(B)** ULIF vs. PLIF.

The Visual Analog Scale score for back pain at the final follow-up could be obtained in six studies (29, 31–35), and no substantial heterogeneity was detected (*I*^2^ = 0.0%, *P* = 0.995). The pooled results revealed no significant difference in VAS-BP at the final follow-up between the ULIF group and the MI-TLIF group (WMD, −0.12; 95% CI −0.25–0.01, *P* = 0.069; Figure 7A).

##### ULIF vs. PLIF

The Visual Analog Scale score for back pain at the final follow-up could be obtained in three studies (36, 38, 39), and no substantial heterogeneity was detected (*I*^2^ = 0.0%, *P* = 0.574). The pooled results revealed no significant difference in VAS-BP at the final follow-up between the ULIF group and the PLIF group (WMD, −0.17; 95% CI −0.37–0.04, *P* = 0.114; Figure 7B).

### Visual Analog Scale score for leg pain

#### One to two weeks post-operatively

##### ULIF vs. MI-TLIF

The Visual Analog Scale score for leg pain at 1–2 weeks post-operatively could be obtained in two studies (31, 35), and no substantial heterogeneity was detected (*I*^2^ = 0.0%, *P* = 1.000). The pooled results revealed no significant difference in VAS-LP at 1–2 weeks post-operatively between the ULIF group and the MI-TLIF group (WMD, −0.20; 95% CI −0.56–0.16, *P* = 0.281).

##### ULIF vs. PLIF

The Visual Analog Scale score for leg pain at 1–2 weeks post-operatively could be obtained in two studies (38, 39), and no substantial heterogeneity was detected (*I*^2^ = 0.0%, *P* = 0.465). The pooled results revealed significantly lower VAS-LP at 1–2 weeks post-operatively in the ULIF group compared with that in the PLIF group (WMD, −0.40; 95% CI −0.72 to −0.08, *P* = 0.014).

#### One month post-operatively

##### ULIF vs. MI-TLIF

The Visual Analog Scale score for leg pain at 1 month post-operatively could be obtained in four studies (29, 31, 34, 35), and no substantial heterogeneity was detected (*I*^2^ = 0.0%, *P* = 0.592). The pooled results revealed no significant difference in VAS-LP at 1 month post-operatively between the ULIF group and the MI-TLIF group (WMD, −0.15; 95% CI −0.34–0.03, *P* = 0.100).

#### The sixth month post-operatively

##### ULIF vs. MI-TLIF

The Visual Analog Scale score for leg pain at 6 month post-operatively could be obtained in two studies (32, 34), and no substantial heterogeneity was detected (*I*^2^ = 0.0%, *P* = 0.710). The pooled results revealed no significant difference in VAS-LP at 6 month post-operatively between the ULIF group and the MI-TLIF group (WMD, 0.49; 95% CI −0.02–1.00, *P* = 0.059).

#### Final follow-up

##### ULIF vs. MI-TLIF

**Figure 8:**
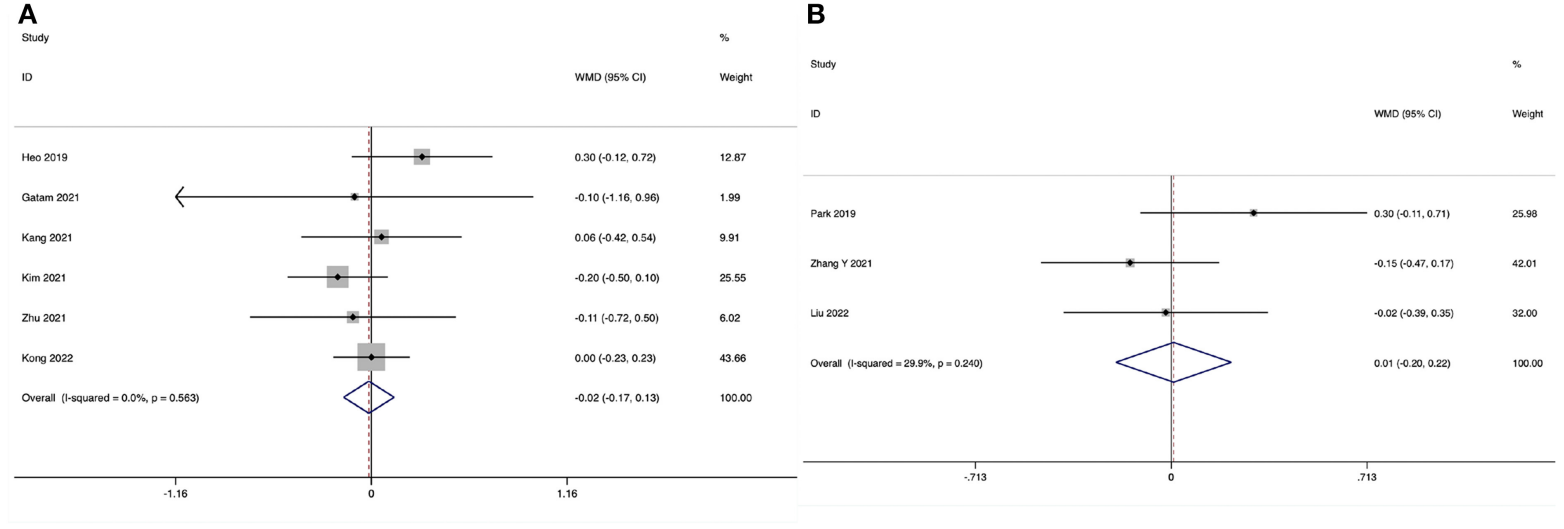
Forest plot of the Visual Analog Scale score for leg pain at the final follow-up. **(A)** ULIF vs. MI-TLIF; **(B)** ULIF vs. PLIF.

The Visual Analog Scale score for leg pain at the final follow-up could be obtained in six studies (29, 31–35), and no substantial heterogeneity was detected (*I*^2^ = 0.0%, *P* = 0.563). The pooled results revealed no significant difference in VAS-LP at the final follow-up between the ULIF group and the MI-TLIF group (WMD, −0.02; 95% CI −0.17–0.13, *P* = 0.843; Figure 8A).

##### ULIF vs. PLIF

The VAS-BP at the final follow-up could be obtained in three studies (36, 38, 39), and no significant heterogeneity was detected (*I*^2^ = 29.9%, *P* = 0.240). The pooled results revealed no significant difference in VAS-LP at the final follow-up between the ULIF group and the PLIF group (WMD, 0.01; 95% CI −0.20–0.22, *P* = 0.937; Figure 8B).

### Excellent/good rate of modified Macnab criteria

#### ULIF vs. MI-TLIF

The excellent/good rate of surgical therapy according to the modified Macnab criteria at the final follow-up could be obtained in three studies (30, 31, 35), and no substantial heterogeneity was detected (*I*^2^ = 0.0%, *P* = 0.856). The pooled results revealed no significant difference in the excellent/good rate of modified Macnab criteria between the ULIF group and the MI-TLIF group (RR, 1.00; 95% CI 0.91–1.11, *P* = 0.951).

#### ULIF vs. PLIF

The excellent/good rate of surgical therapy according to the modified Macnab criteria at the final follow-up could be obtained in two studies (37, 39), and no substantial heterogeneity was detected (*I*^2^ = 0.0%, *P* = 0.561). The pooled results revealed no significant difference in the excellent/good rate of modified Macnab criteria between the ULIF group and the PLIF group (RR, 0.97; 95% CI 0.82–1.14, *P* = 0.709).

### Laboratory outcomes

#### Creatine phosphokinase

##### ULIF vs. MI-TLIF

Post-operative CPK could be obtained in two studies (28, 34), and significant heterogeneity was detected (*I*^2^ = 69.0%, *P* = 0.057). The pooled results revealed significantly lower post-operative CPK in the ULIF group compared with that in the MI-TLIF group (SMD, −1.15; 95% CI −1.86 to −0.45, *P* = 0.001).

#### C-reactive protein

##### ULIF vs. MI-TLIF

Post-operative CRP could be obtained in two studies (28, 34), and no significant heterogeneity was detected (*I*^2^ = 42.3%, *P* = 0.188). The pooled results revealed significantly lower post-operative CRP in the ULIF group compared with that in the MI-TLIF group (SMD, −1.21; 95% CI −1.59 to −0.83, *P* < 0.001).

### Radiographic outcomes

#### Fusion rate

##### ULIF vs. MI-TLIF

**Figure 9:**
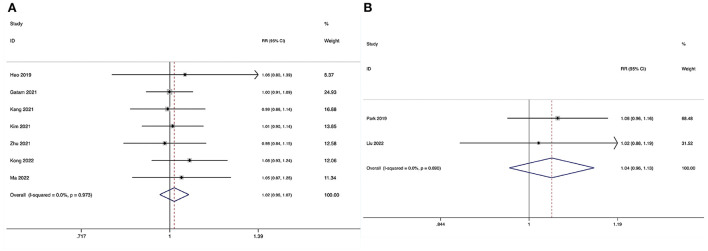
Forest plot of the fusion rate. **(A)** ULIF vs. MI-TLIF; **(B)** ULIF vs. PLIF.

The fusion rate at the final follow-up could be obtained in seven studies (29–35), and no substantial heterogeneity was detected (*I*^2^ = 0.0%, *P* = 0.973). The pooled results revealed no significant difference in fusion rate between the ULIF group and the MI-LIF group (RR, 1.02; 95% CI 0.96–1.07, *P* = 0.545; Figure 9A).

##### ULIF vs. PLIF

The fusion rate at the final follow-up could be obtained in two studies (38, 39), and no substantial heterogeneity was detected (*I*^2^ = 0.0%, *P* = 0.690). The pooled results revealed no significant difference in fusion rate between the ULIF group and the PLIF group (RR, 1.04; 95% CI 0.96–1.13, *P* = 0.296; Figure 9B).

#### Cage subsidence

##### ULIF vs. MI-TLIF

The incidence of cage subsidence at the final follow-up could be obtained in three studies (30, 32, 33), and no substantial heterogeneity was detected (*I*^2^ = 0.0%, *P* = 0.498). The pooled results revealed no significant difference in the incidence of cage subsidence between the ULIF group and the MI-TLIF group (RR, 0.34; 95% CI 0.08–1.46, *P* = 0.146).

#### Unplanned return to the operating room

##### ULIF vs. MI-TLIF

The incidence of unplanned return to OR could be obtained in four studies (32–35), and no substantial heterogeneity was detected (*I*^2^ = 0.0%, *P* = 0.689). The pooled results revealed no significant difference in the incidence of unplanned return to OR between the ULIF group and the MI-TLIF group (RR, 0.76; 95% CI 0.20–2.93, *P* = 0.687).

### Surgical complications

#### Overall

##### ULIF vs. MI-TLIF

**Figure 10:**
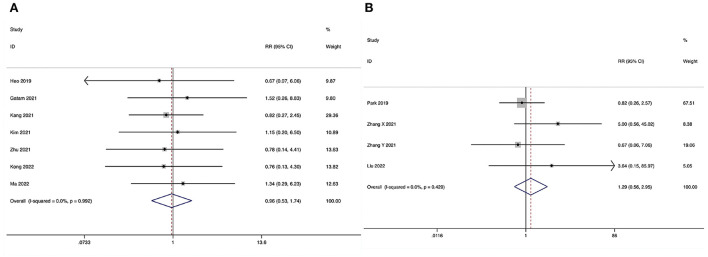
Forest plot of the overall surgical complication rate. **(A)** ULIF vs. MI-TLIF; **(B)** ULIF vs. PLIF.

The overall surgical complication rate during the perioperative period could be obtained in seven studies (29–35), and no substantial heterogeneity was detected (*I*^2^ = 0.0%, *P* = 0.992). The pooled results revealed no significant difference in the overall surgical complication rate between the ULIF group and the MI-TLIF group (RR, 0.96; 95% CI 0.53–1.74, *P* = 0.896; Figure 10A).

##### ULIF vs. PLIF

The overall surgical complication rate during the perioperative period could be obtained in four studies (36–39), and no substantial heterogeneity was detected (*I*^2^ = 0.0%, *P* = 0.429). The pooled results revealed no significant difference in the overall surgical complication rate between the ULIF group and the PLIF group (RR, 1.29; 95% CI 0.56–2.95, *P* = 0.553; Figure 10B).

#### Epidural hematoma

##### ULIF vs. MI-TLIF

The incidence of epidural hematoma could be obtained in five studies (29, 31, 33–35), and no substantial heterogeneity was detected (*I*^2^ = 0.0%, *P* = 0.951). The pooled results revealed no significant difference in the incidence of an epidural hematoma between the ULIF group and the MI-TLIF group (RR, 1.19; 95% CI 0.37–3.83, *P* = 0.775).

#### Dural tear

##### ULIF vs. MI-TLIF

**Figure 11:**
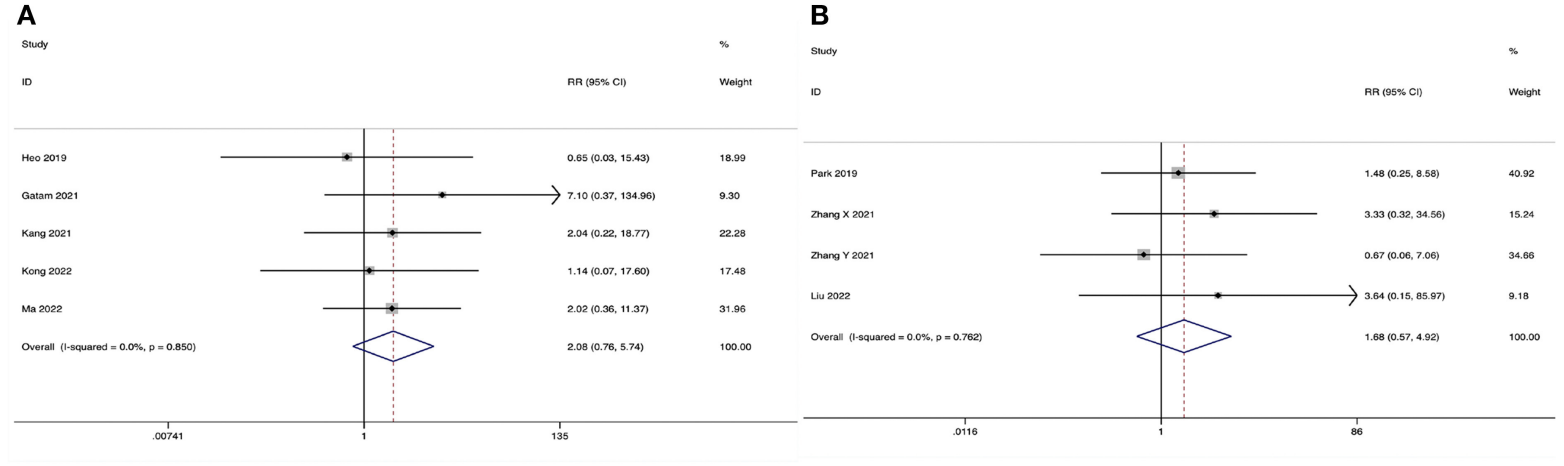
Forest plot of the incidence of dural tear. **(A)** ULIF vs. MI-TLIF; **(B)** ULIF vs. PLIF.

The incidence of dural tear could be obtained in five studies (29, 30, 32–34), and no substantial heterogeneity was detected (*I*^2^ = 0.0%, *P* = 0.850). The pooled results revealed no significant difference in the incidence of dural tear between the ULIF group and the MI-TLIF group (RR, 2.08; 95% CI 0.76–5.74, *P* = 0.156; Figure 11A).

##### ULIF vs. PLIF

The incidence of dural tear could be obtained in four studies (36–39), and no substantial heterogeneity was detected (*I*^2^ = 0.0%, *P* = 0.762). The pooled results revealed no significant difference in the incidence of dural tear between the ULIF group and the PLIF group (RR, 1.68; 95% CI 0.57–4.92, *P* = 0.344; Figure 11B).

#### Surgical site infection

##### ULIF vs. MI-TLIF

The incidence of surgical site infection could be obtained in five studies (29, 30, 32–34), and no substantial heterogeneity was detected (*I*^2^ = 0.0%, *P* = 0.985). The pooled results revealed no significant difference in the incidence of surgical site infection between the ULIF group and the MI-TLIF group (RR, 0.34; 95% CI 0.09–1.33, *P* = 0.120).

#### Neurologic deficits

##### ULIF vs. MI-TLIF

The incidence of neurologic deficits could be obtained in three studies (31, 34, 35), and no substantial heterogeneity was detected (*I*^2^ = 0.0%, *P* = 0.721). The pooled results revealed no significant difference in the incidence of neurologic deficits between the ULIF group and the MI-TLIF group (RR, 0.74; 95% CI 0.22–2.50, *P* = 0.632).

##### ULIF vs. PLIF

The incidence of neurologic deficits could be obtained in two studies (37, 38), and no significant heterogeneity was detected (*I*^2^ = 28.5%, *P* = 0.237). The pooled results revealed no significant difference in the incidence of neurologic deficits between the ULIF group and the PLIF group (RR, 1.25; 95% CI 0.20–7.64, *P* = 0.811).

### Sensitivity analyses

The sensitivity analyses indicated that the additional omission of any study would not significantly affect the results, which verified the stability of the data and rationality of the analyses.

## Discussion

Enhanced Recovery After Surgery is a multidisciplinary perioperative care pathway designed to achieve early recovery for patients undergoing major surgery. The three phases of ERAS protocol are pre-operative, intraoperative, and post-operative periods, and the key components include optimization of nutrition, emotional support, multimodal opioid-sparing analgesia, antimicrobial prophylaxis, appropriate surgical procedure, and early mobilization (40). Since the first publication of the ERAS consensus statement in 2005, the ERAS Society has now published guidelines in more than 20 surgical specialties, including colorectal surgery (11), pancreatoduodenectomy (41), radical cystectomy (42), gastrectomy (43), bariatric surgery (44), liver surgery (45), lung surgery (46), and cardiac surgery (47). For spine surgery, some cohort studies and a meta-analysis suggested that improved outcomes could be obtained through the implementation of ERAS protocols during the perioperative period (48–51). In 2021, an evidence-based recommendation for lumbar fusion surgery was developed by the ERAS Society (52). Although surgical techniques should be decided on a case-by-case basis, the minimally invasive technique achieved a strong recommendation grade because is paramount for post-operative recovery (52, 53).

Conventional MI-TLIF and open PLIF were effective surgical procedures of lumbar interbody fusion for treating LDD, but the paraspinal muscle damage and blood loss may delay pain relief and functional recovery. With the advancement of optical technologies, water-based endoscopic procedures have gained popularity (54–57). ULIF, combining the endoscope and the minimally invasive spine instruments, has been increasingly used as an alternative to conventional lumbar interbody fusion techniques (18, 58). This systematic review and meta-analysis directly compared the outcomes and complications of ULIF to conventional MI-TLIF or PLIF for LDD. Different from the previous meta-analysis by Lin et al. the current study did not merge the patients who underwent MI-TLIF or PLIF in a single group because these two posterior procedures had very different paraspinal muscle injury levels (59). The results revealed that there was no significant difference in the radiographic outcomes and complications between the ULIF group and MI-TLIF or PLIF group. Nevertheless, enhanced recovery was observed through superior clinical outcomes, surgical data, and laboratory outcomes in patients receiving ULIF.

The LOS was shortened by 1.27 days (*P* < 0.001) and 4.40 days (*P* = 0.018) in the ULIF group compared with the MI-TLIF group and the PLIF group, respectively. This effect was associated with enhanced pain relief and function recovery by ULIF. The current study suggests that ULIF has a significantly better short-term improvement in VAS-BP and ODI than both MI-TLIF and PLIF groups. In addition, an enhanced short-term improvement in VAS-LP and long-term improvement in ODI were noted in the ULIF group compared with the PLIF group. These findings may be attributed to the reduced atrophy, denervation, and ischemic paraspinal muscle damage caused by dissection and retraction (60, 61). Furthermore, the endoscope provides a clear and magnified view, allowing more precise manipulation for the decompression of the central canal, lateral recess, and bilateral nerve roots (32). The study by Kim et al. reported that there was no significant difference in the early and final ODI between unilateral biportal endoscopic and open microscopic techniques for lumbar discectomy (61). Therefore, preserving paraspinal muscle and posterior soft tissue may benefit lumbar interbody fusion more than sole discectomy, which requires less muscle retraction. Inevitable systemic inflammatory response due to iatrogenic muscle injury is associated with post-operative pain and disability (34, 62). Thus, effective alleviation or suppression of the inflammatory response is essential for the enhanced recovery of patients. CPK and CRP were presentative biomarkers, which peeked on post-operative 2–3 days, recovering to the normal range weeks after the surgery (39, 63). In the current study, the peek of both CPK and CRP was significantly lower in the ULIF group, indicating that the biportal endoscopic technique produces less systemic inflammatory response than conventional procedures. This advantage may also relate to favorable pain relief, function improvement, and LOS.

Like conventional procedures, ULIF is also frequently accompanied by substantial surgical blood loss, especially when resecting ligamentum flavum and superior articular process, which would postpone the recovery and induce complications (63, 64). In this study, we found that the EBL and post-operative drainage in the ULIF group were significantly reduced than both the MI-TLIF group and the PLIF group. Continuous fluid irrigation played a vital role in controlling epidural and bone surface hemorrhage. However, the pressure of irrigation and constant outflow should be noted to prevent post-operative neck pain and seizures caused by increased intracranial pressure (65). In addition, rather than electrocautery, bipolar radiofrequency ablation could be applied to obtain effective microvascular coagulation around the dural sac (34, 38, 66). Therefore, better bleeding control leads to less post-operative drainage and early mobilization in patients who underwent ULIF.

Evaluating the fusion rate is of paramount importance for patients who underwent lumbar interbody fusion, as failed solid fusion could jeopardize the surgical effect and quality of life (67). This study yielded similar fusion rates between ULIF and conventional procedures (ULIF vs. MI-TLIF, 89.8 vs. 87.7%; ULIF vs. PLIF, 94.3 vs. 90.3%). Some advantages of the biportal endoscopic system might facilitate the fusion rate of ULIF. Meticulous endplate preparation could be performed under the clean and magnified real-time surgical visualization, offering a favorable fusion environment by completely removing the cartilaginous portion (35, 68). Continuous fluid irrigation may disperse the thermal energy, which could induce necrosis of the endplate and further cage subsidence (34, 69). Moreover, unlike the uniportal endoscopic system which only allows small-sized cages to pass the cannula, large-sized or expandable cages could be used in an independent working portal, which obtained a favorable fusion rate as conventional procedures.

Although there are various advantages, longer ORT and a steep learning curve are the potential drawbacks of ULIF. In the current study, the ORT of the ULIF group was 179.63 ± 29.34 min in MI-TLIF studies and 184.43 ± 41.50 min in PLIF studies, which were significantly longer than the 148.01 ± 24.17 min in the MI-TLIF group and 130.87 ± 23.22 min of the PLIF group. The biportal endoscopic technique is just like arthroscopy. During the initial stages of the learning curve, single-handed instrument handling and identification of anatomical landmarks may be factors that increase the ORT for ULIF (38). For less-experienced surgeons, delicate decompression manipulations may become complex and easily induce complications. Although the surgical complication rate was similar between ULIF and conventional procedures (ULIF vs. MI-TLIF, 6.9 vs. 6.7%; ULIF vs. PLIF, 6.8% vs. 5.1%), the slightly higher incidence of dural tear in the ULIF group should be noted (ULIF vs. MI-TLIF, 4.8% vs. 2.1%; ULIF vs. PLIF, 4.7% vs. 2.8%). Therefore, ULIF is recommended for surgeons who have performed at least 54 cases of biportal endoscopic decompression (70). Another complication was the epidural hematoma, most likely due to oozing from the bone trapped under the intact posterior tension band, which usually could be resolved by itself (32). The incidence of this complication was low (ULIF vs. MI-TLIF, 2.9 vs. 2.3%). The incidence of surgical site infection was slightly lower in the ULIF group than in the MI-TLIF group (0.0 vs. 2.6%). This finding may be attributed to the reduced surgical smoke and wound contamination by the bipolar radiofrequency ablation.

## Limitations

This study has several limitations. First, the impact of smoking was not considered due to the missing data, which could overestimate the fusion rate and cage subsidence rate (71). Second, most studies lacked data on comorbidities, which could have influenced some of the outcomes analyzed. Third, the heterogeneity of the included patients should be acknowledged because they had various LDD, including LSS, LDH, and LS. Fourth, the number of research studies focused on the comparison of ULIF and conventional procedures is still limited. Therefore, some pooled outcomes may not be reliable when more results were reported in future studies. Additionally, no randomized controlled study was included at a higher level of methodological quality. Further multicenter randomized controlled trials with longer follow-up periods should be performed to obtain more convincing conclusions.

## Conclusion

Compared with conventional MI-TLIF and PLIF, ULIF was associated with reduced EBL, shorter LOS, alleviated inflammatory response, and comparable fusion rate as well as complication management. Compared with MI-TLIF, a better short-term improvement in VAS-BP and ODI was achieved by ULIF; compared with open PLIF, additional enhanced short-term improvement in VAS-LP and long-term improvement in ODI were observed in ULIF. ULIF could enhance the recovery of patients with LDD compared with conventional posterior procedures.

## Data availability statement

The raw data supporting the conclusions of this article will be made available by the authors, without undue reservation.

## Author contributions

HY, YL, and AP contributed to the study concept and design, revised, and edited the manuscript. HY, FC, and YL took part in the initial literature search and assessed the eligibilities of feasible studies. HY and FC interpreted the findings and wrote the first draft of the manuscript. HY, FC, and AP prepared the figures and tables. All authors approved the final version of the manuscript, contributed to the article, and approved the submitted version.
